# PD-L1 is associated with the prognosis of penile cancer: A systematic review and meta-analysis

**DOI:** 10.3389/fonc.2022.1013806

**Published:** 2022-11-30

**Authors:** Yi Lu, Yutao Wang, Hao Su, Hongjun Li

**Affiliations:** Department of Urology, Peking Union Medical College Hospital, Peking Union Medical College, Chinese Academy of Medical Sciences, Beijing, China

**Keywords:** PD-L1, immune checkpoint, prognostic biomarker, meta-analysis, penile carcinoma

## Abstract

**Background:**

Previous studies have explored the role of PD-L1 in the survival outcomes of penile cancer patients with controversies existed. Thus, the meta-analysis was conducted to report and review the association between PD-L1 and survival in penile cancer patients.

**Methods:**

PubMed, Cochrane Library, EMBASE, and Web of Science were all searched, screened, and reviewed by June 1, 2022. Hazard ratio (HR) was used to evaluate the relationship between PD-L1 and survival outcome, and odds ratio (OR) was for tumor features.

**Results:**

Nine retrospective studies (1,003 patients) were incorporated. The prevalence of PD-L1 in patients with penile cancer was 51.4% (95% CI = 42.1%-60.8%, *I* ^2^ = 88.5%). Higher PD-L1 on tumor cells was related to shorter cancer-specific survival (CSS) in patients (HR = 1.578, 95% CI = 1.227-2.029, *I* ^2^ = 23.3%), but had no associations with overall survival (OS) (HR = 1.123, 95% CI = 0.511-2.465, *I* ^2^ = 0.0%). Subgroup analysis indicated that higher PD-L1 was related to shorter CSS in Caucasus (HR = 1.827, 95% CI = 1.355-2.465, *I* ^2^ = 0.0%) only. Furthermore, PD-L1 had associations with tumor stage (pT1 vs. pT2-4, OR = 0.480, 95% CI = 0.346-0.667, *P* = 0.001) and tumor grade (Well and moderate vs. Poor, OR = 0.377, 95% CI = 0.264-0.538, *P* < 0.001). PD-L1 positivity was also related to lymph node (LN) status (pN0/NX vs. pN1–3, OR = 0.541, 95% CI = 0.385-0.759, *P* = 0.001) and HPV status (Positive vs. Negative, OR = 0.510, 95% CI = 0.322-0.810, *P* = 0.003). A trend toward statistical significance between PD-L1 and histological types was also observed (Usual SCC vs. Others, OR = 1.754, 95% CI = 0.984-3.124, *P* = 0.070).

**Conclusions:**

PD-L1 over-expression was related to worse survival outcomes and several clinicopathological features of penile cancer. PD-L1 expression can be applied to select appropriate treatment strategies for penile malignancies.

**Systematic review registration:**

https://www.crd.york.ac.uk/prospero/display_record.php?RecordID=343041, identifier CRD42022343041.

## Introduction

Penile cancer, with substantial differences in prevalence, is a rare carcinoma in industrialized countries (1). Penile squamous cell carcinoma (SqCC) accounts for 95% of all penile malignancies (2). Therapeutic options for locally advanced metastatic penile SqCC are limited and most of the patients die within one year (3). Surgery combined with subsequent platinum-based chemotherapy usually had a moderate response, while 63.3% of patients still suffer tumor recurrence or progression after first-line chemotherapy (paclitaxel, ifosfamide, and cisplatin) and the median survival is only 5.6 months (4). Recently, immunotherapy has yielded dramatically improved long-term survival benefits in several SqCC, especially among patients with high programmed death receptor ligand-1 (PD-L1)/programmed death receptor-1 (PD-1) expression (5, 6). Given the high expression of PD-L1 in the penile SqCC tumor microenvironment, immunotherapy may be an efficient strategy (7).

The immune checkpoint had been regarded as the milestone event in tumor research for decades. The mechanisms underlying tumor development and progression in the background of immunotherapy have also been widely discussed (8, 9). Recently, the favorable efficacy of immunotherapy has been found and validated in many malignancies (10–12). PD-L1, on the tumor cells, could bind to PD-1, suppressing immune cell proliferation and release of immune molecules. Tumor cells can evade immune surveillance through immune checkpoints. Finally, tumor recurrence or metastasis happened (13, 14). In the field of penile cancer, increasing studies have demonstrated the role of PD-L1 expression in survival outcomes and they got controversial findings. Some evidence indicated that higher PD-L1 was related to poor survival for penile cancer (15), while some reported opposing findings (16, 17). Therefore, we formulated clinical questions under the guidance of the PICOS strategy and firstly assessed the role of PD-L1 expression (high or low) in survival outcomes and the clinicopathological features in penile cancer patients through a meta-analysis.

## Methods

### Data sources

Detailed inclusion criteria were raised according to the established reporting guidelines (18, 19). Three authors independently reviewed all available literature in PubMed, Cochrane Library, EMBASE, and Web of Science in June 2022. No eligible randomized-controlled trials (RCTs) with interventions were found and observational studies reporting the effect of PD-L1 on tumor behaviors or survival outcomes in penile cancer patients were all included. The references and citations were also searched and checked carefully. The keywords for the search were “PD-L1” and “penile cancer”. **Supplementary Table 1** showed the detailed search strategy. Notably, this study is a conventional trial-level meta-analysis, thus no individual patient-level data were available. The protocol of the study was registered in PROSPERO (CRD42022343041).

### Criteria for inclusion and exclusion

Inclusion criteria: (a) Population: penile cancer patients without non-surgical treatments. (b) Interventions: Expression of PD-L1 (≥ cut-off value) on tumor cells. (c) Comparators: Expression of PD-L1 (< cut-off value) on tumor cells. (d) Outcomes: Survival outcomes or clinicopathological characteristics of penile carcinoma cases. (e) Study design: No restriction. (f) Article types: Original article or study with standard reporting and sufficient data. (g) Information on survival outcomes: Hazard ratio (HR) and 95% confidence interval (95%CI) could be obtained directly or indirectly. (i) Studies with a sample size of more than 20. We excluded studies that can’t meet the inclusion criteria or those with low quality for reporting.

### Data collection

Three authors screened the retrieved records independently. Items including the first author, study year, study design, study region, demographic information, cut-off value, median follow-up duration, and survival outcomes were extracted. We obtained missing or unclear information through contact with the article authors. Information will be considered as not mentioned or not available if there was no reply. By using the validated tool (20), HRs and their 95%CIs were digitized from studies that only had Kaplan-Meier curves.

### Risk of bias assessment

A modified Newcastle–Ottawa scale (NOS) was used for RoB analysis (21). An agreement was reached through consensus among the 3 authors and communication with the article authors.

### Statistical analysis

PD-L1 expression and its association with tumor behaviors were presented by pooled odds ratio (ORs) and HR was used to demonstrate the relationship between PD-L1 and survival outcomes. If significant heterogeneity was found or *I* ^2^ > 50%, we utilized random-effect models, otherwise we chose fixed-effect models (22). Publication bias was evaluated through Begg’s test and Egger’s test, and displayed by funnel plots. Sensitivity analyses were done by excluding a study at one time and a cumulative meta-analysis was also done. STATA 12.0 (Stata-Corp.) was used for statistical analyses. A two-tailed *P* < 0.05 was considered statistically significant.

## Results

### Literature selection

**Figure 1:**
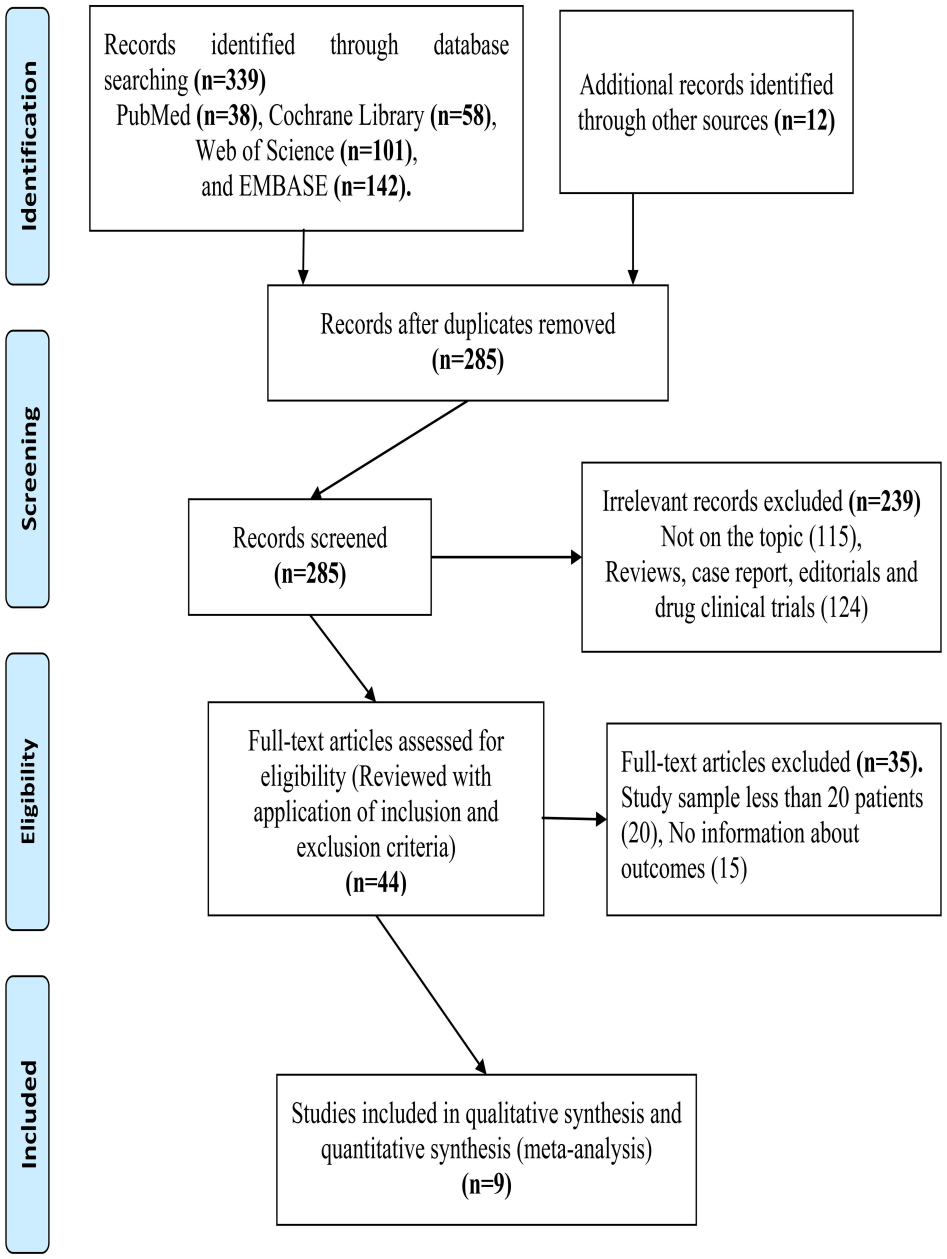
PRISMA flow chart of the the data search.

Two hundred and eighty-five non-repeated records were identified. We excluded records for the following reasons: not original articles or irrelevant topics (n=239), studies with limited sample size (≤ 20) (n=20), or insufficient data (n=17). Finally, we included 9 retrospective cohort studies (1,003 individuals) in the study (shown in **Figure 1**) (15–17, 22–27).

### Characteristics of included studies

All 9 studies were published in recent six years (**Table 1**) and they were conducted across 6 countries, with 3 studies in China, 2 in the USA, 1 in Brazil, 1 in Sweden, 1 in the Netherlands, and 1 in Germany. Immunohistochemistry (IHC) was adopted to analyze the expression of PD-L1 in tumor tissues in all the studies. All studies had NOS grades ≥ 7 (**Supplementary Table 2**).

**Table 1:** Characteristics of included studies.

Study (author, year) [reference]	Study type	Study region	Ethnicity	Sample size	Cutoff value	Positive PD-L1 (%)	Median follow-up (months)	Survival	Evaluated cells	Method
Udager 2016 (15)	RC	USA	Caucasian	37 penile Sqcc	≥5%	62.16	N.M.	CSS	Tumor cells and TILs	Immunohistochemistry (Antibody: clone 5H1)
Cocks 2016 (22)	RC	USA	Caucasian	53 penile Sqcc	N.M.	39.62	N.M.	CSS/OS	Tumor cells	Immunohistochemistry (Antibody: E1L3N)
Ottenhof 2016 (24)	RC	Netherlands	Caucasian	213 penile cancer	≥1%	48	62	CSS	Tumor cells and TILs	Immunohistochemistry (Antibody: E1L3N)
Deng 2017 (23)	RC	China	Asian	116 penile Sqcc	≥5%	53.4	N.M.	CSS	Tumor cells and TILs	Immunohistochemistry (Antibody: E1L3N)
Davidsson 2018 (25)	RC	Sweden	Caucasian	222 penile Sqcc	≥5%	31.53	34	CSS	Tumor cells and TILs	Immunohistochemistry (Antibody: SP142 and 28.8)
De Bacco 2019 (26)	RC	Brazil	Caucasian	40 penile Sqcc	≥1%	51.4	46.87	OS	Tumor cells	Immunohistochemistry (Antibody: colne ZR3, zeta-corporation)
Chu 2020 (16)	RC	China	Asian	178 penile Sqcc	≥1%	67.42	88	CSS	Tumor cells and TILs	Immunohistochemistry (Antibody: E1L3N)
Hu 2020 (17)	RC	China	Asian	84 penile Sqcc	H-Score	60.71	32.1	CSS	Tumor cells	Immunohistochemistry (Antibody: ab205921, Abcam)
Müller 2022 (27)	RC	Germany	Caucasian	60 penile Sqcc	N.M.	50	N.M.	OS	Tumor cells and TILs	Immunohistochemistry (Antibody: clone ZR3)

RC, retrospective cohort study; OS, overall survival; CSS, cancer specific survival; IHC, immunohistochemistry staining; N.M, not mentioned; TIL, tumor infiltrating lymphocytes.

### Prevalence of PD-L1 in penile carcinoma

**Figure 2:**
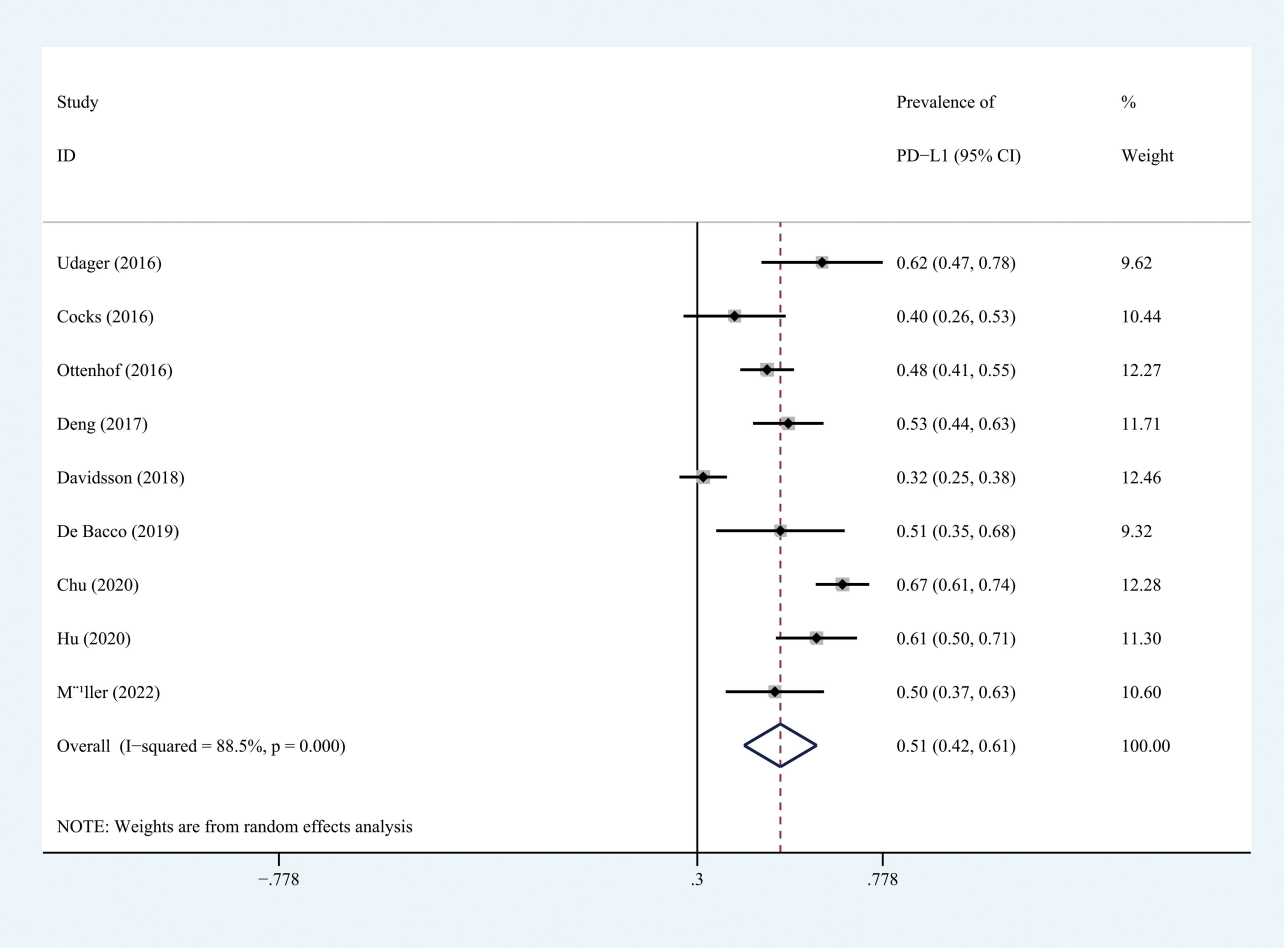
Prevalence of PD-L1 expression in penile cancer. CI, confidence interval.

The prevalence ranges from 48.0 to 67.4% in studies (**Table 1**). The pooled prevalence of PD-L1 in penile cancer was 51.4% (random effect, 95%CI=42.1-60.8%, *I* ^2^ = 88.5%) (**Figure 2**).

### Association between PD-L1 and survival

**Figure 3:**
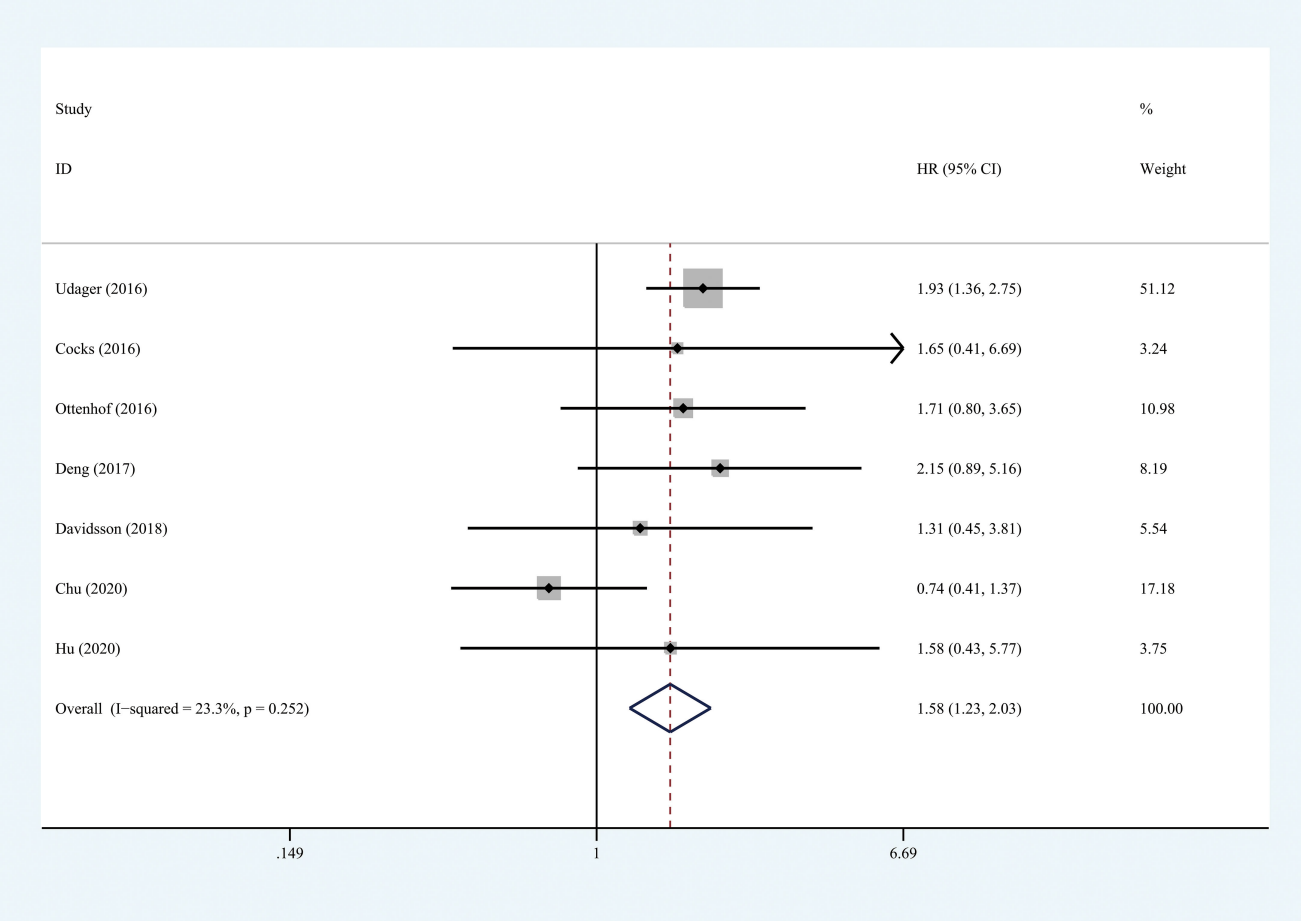
Prognostic value of PD-L1 for CSS. CSS, cancer specific survival; HR, hazard ratio; CI, confidence interval.

**Figure 4:**
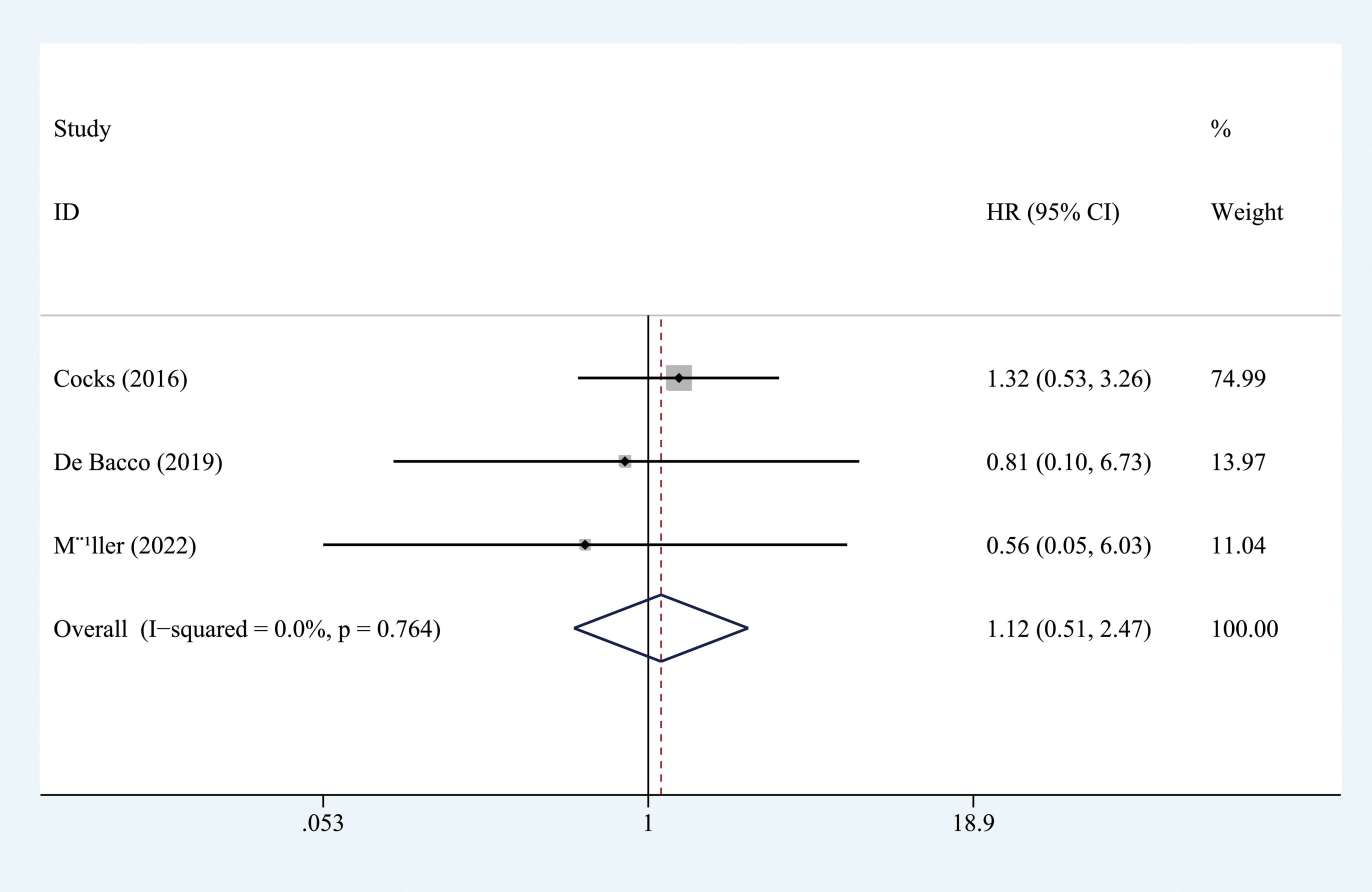
Prognostic value of PD-L1 for OS. OS, overall survival; HR, hazard ratio; CI, confidence interval.

Seven studies, with 903 individuals, reported CSS (summarized in **Table 2**). The pooled results demonstrated that higher PD-L1 level was associated with shorter CSS (HR = 1.578, 95% CI = 1.227-2.029, *I* ^2^ = 23.3%) (**Figure 3**). Three studies, with 153 individuals, reported OS. We found that PD-L1 had no significant association with OS in patients with penile carcinoma (HR = 1.123, 95% CI = 0.511-2.465, *I* ^2^ = 0.0%) (**Figure 4**).

**Table 2:** Subgroup analyses between PD-L1 expression and survival outcomes.

	OS					CSS				
	No. of studies	Pooled HR(95% CI)	Heterogeneity		*P*-value for interaction	No. of studies	Pooled HR(95% CI)	Heterogeneity		*P*-value for interaction
			*I* ^2^ (%)	P-value				*I* ^2^ (%)	P-value
Overall	3	1.123 (0.511, 2.465)	0.0	0.764		7	1.578 (1.227, 2.029)	23.3	0.252	
Year of publication					0.523					0.130
2018 and before	1	1.320 (0.532, 3.274)	—	—		5	1.868 (1.400, 2.465)	0.0	0.959	
After 2018	2	0.691 (0.143, 3.331)	0.0	0.824		2	0.852 (0.491, 1.475)	5.8	0.303	
Race					0.253					0.075
Caucasus	3	1.123 (0.511, 2.465)	0.0	0.764		4	1.827 (1.355, 2.462)	0.0	0.914	
Asian	0	—	—	—		3	1.105 (0.693, 1.761)	51.6	0.127	
NOS score					0.485					0.100
7	2	1.184 (0.507, 2.764)	0.0	0.512		2	1.915 (1.362, 2.693)	0.0	0.829	
8	1	0.810 (0.099, 6.645)	—	—		5	1.253 (0.863, 1.818)	21.0	0.281	
Region					0.326					0.156
USA	1	1.320 (0.532, 3.274)	—	—		2	1.915 (1.362, 2.693)	0.0	0.829	
Netherland	0	—	—	—		1	1.710 (0.801, 3.653)	—	—	
China	0	—	—	—		3	1.105 (0.693, 1.761)	51.6	0.127	
Sweden	0	—	—	—		1	1.310 (0.450, 3.812)	—	—	
Brazil	1	0.810 (0.099, 6.645)	—	—		0	—	—	—	
Germany	1	0.565 (0.053, 6.028)	—	—		0	—	—	—	

OS, overall survival; CSS, cancer specific survival.

Results of meta-regression and subgroup analyses were presented in **Table 2**. No significant results were determined among the subgroups in terms of OS. The subgroup analysis by race indicated that higher PD-L1 (higher than cut-off values) was associated with shorter CSS in Caucasians (HR = 1.827, 95% CI = 1.355-2.465, *I* ^2^ = 0.0%) but not in Asians (HR = 0.852, 95% CI = 0.491-1.475, *I* ^2^ = 5.8%). Moreover, results indicated that higher PD-L1 levels were associated with shorter CSS in the studies conducted in the USA (HR = 1.915, 95% CI = 1.362-2.693, *I* ^2^ = 0.0%), but not in other countries. No significant difference was determined in any subgroup (*P*_interaction_ > 0.05 for all).

### PD-L1 and tumor behaviors

Results on this were recorded in **Table 3**. We observed that higher PD-L1 in penile cancer had relationships with higher tumor stage (pT1 vs. pT2-4, OR = 0.480, 95% CI = 0.346-0.667, *P* = 0.001) and more advanced tumor grade (Well and moderate vs. Poor, OR = 0.377, 95% CI = 0.264-0.538, *P* < 0.001). Significant associations were also found between PD-L1 positivity and lymph node (LN) positivity (pN0/NX vs. pN1–3, OR = 0.541, 95% CI = 0.385-0.759, *P* = 0.001) and HPV negativity (Positive vs. Negative, OR = 0.510, 95% CI = 0.322-0.810, *P* = 0.003). Moreover, there was a trend toward statistical significance between PD-L1 positivity and usual histological types (Usual SCC vs. Others, OR = 1.754, 95% CI = 0.984-3.124, *P* = 0.07). However, PD-L1 levels had no significant associations with penile cancer in terms of lympho-vascular invasion (LVI) (presence vs. absence, OR = 1.005, 95% CI = 0.561-1.800, *P* = 0.087). Local recurrence, distant progression, and p16 were only reported in a single article, thus pooled results were not achievable (15).

**Table 3:** Association between PD-L1 expression and clinicopathological features of penile carcinoma.

Items	No.of studies	Pooled OR (95%CI)	I2 (%)	*P*-value^#^	Model
Grade (Well and moderate vs. Poor)	6	0.377 (0.264, 0.538)	0.0	0.519	Fixed
Lymphovascular invasion (Presence vs. Absence)	3	1.005 (0.561, 1.800)	29.5	0.242	Fixed
Stage (pT1 vs. pT2-4)	6	0.480 (0.346, 0.667)	0.0	0.509	Fixed
LN status (pN0/NX vs. pN1–3)	7	0.541 (0.385, 0.759)	0.0	0.543	Fixed
Histology (Usual SCC vs. Other)	4	1.754 (0.984, 3.124)	2.2	0.381	Fixed
HPV status (Positive vs. Negative)	3	0.510 (0.322, 0.810)	0.0	0.903	Fixed

OR, odds ratio; CI, confidence interval.

^#^P-value of heterogeneity test.

### Publication bias

**Figure 5:**
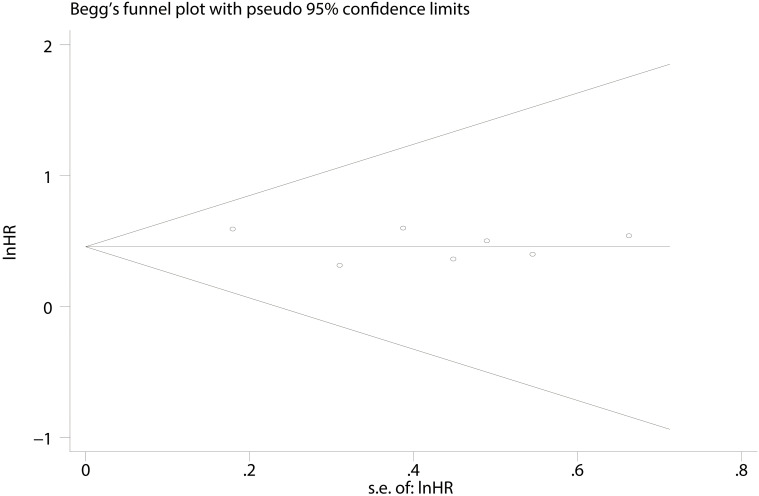
Funnel plots based on PD-L1 for cancer-specific survival.

No significant publication bias was found (CSS: Begg’s test, *P * = 0.652; Egger’s test, *P* = 0.764 (**Figure 5**).

### Sensitivity analysis

**Figure 6:**
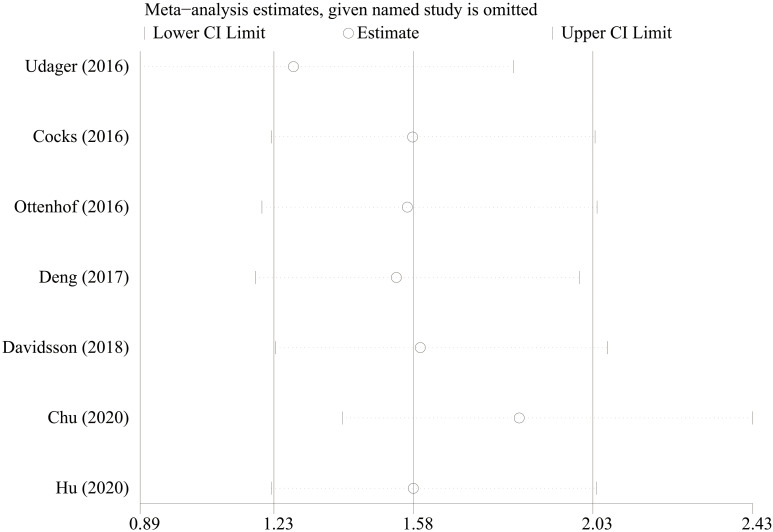
Sensitivity analysis based on PD-L1 for cancer-specific survival.

**Figure 7:**
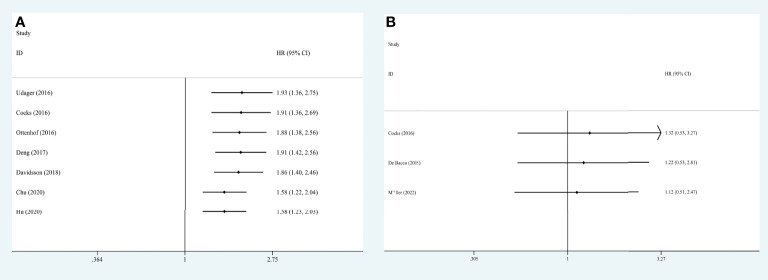
Cumulative meta-analysis for OS **(A)** and CSS **(B)**, based on year of publication. OS, overall survival; CSS, cancer specific survival; HR, hazard ratio; CI, confidence interval.

We extracted each study subsequently in each analysis, finding that no study could affect the pooled result significantly, thus the results were reliable (**Figure 6**). The cumulative meta-analysis was performed in the order of publication year (**Figure 7**). It revealed that higher PD-L1 levels were related to shorter CSS, but not the OS. Furthermore, we also found that the 95%CIs narrowed and the pooled results gradually moved near the null.

## Discussion

To our knowledge, we firstly assessed the role of PD-L1 in penile cancer patients by conducting a meta-analysis. The pooled prevalence of PD-L1 in patients with penile carcinoma was 51.4% (42.1% to 60.8%) and results indicated that higher PD-L1 was not related to OS but was associated with shorter CSS. Notably, higher PD-L1 had associations with worse CSS in Caucasian penile cancer patients, especially patients from the USA. When it comes to tumor behavior, higher PD-L1 had associations with higher tumor stage and grade of penile carcinoma, LN positivity, and HPV negativity. Moreover, a trend toward statistical significance was also observed between positive PD-L1 and usual SqCC.

In the meta-analysis, seven studies reported a high expression rate (>40%) of PD-L1 (15–17, 23, 24, 26, 27), which provided rationality for immunotherapy application in such cancer. However, different cut-off values, small sample sizes, and diverse primary antibody species may influence the prevalence.

Due to the rarity of penile carcinoma, it had been largely neglected by urologic academic associations (28). Up till now, studies reporting the role of PD-L1/PD-1 in the prognosis of urological tumors had been focused mainly on urothelial carcinoma of the bladder (UCB), prostate cancer, and RCC (29). Only the well-designed studies included in the meta-analysis investigated the role of PD-L1 in penile carcinoma, but these studies were often designed differently. The meta-analysis only pooled the results from 9 studies reporting PD-L1 levels from tumor cells. Previous large clinical trials firstly explored the role of immunotherapy in advanced penile cancer or metastatic penile cancer (NCT03333616 and NCT03774901) (30, 31). They showed that these refractory conditions can’t achieve prognostic improvement after first-line modality treatment, while Avelumab or the combination of Nivolumab and Ipilimumab can enhance the prognosis. However, they did not compare the efficacy difference between subgroups of PD-L1 high and low, as researchers did in other types of tumors. Under the light of clinical trials, a few case series and case reports were published, promoting the application of immunotherapy in penile carcinoma and indicating the crucial role of PD-L1 expression in immunotherapy (32–35). Unfortunately, there were few studies in this area, and more well-designed studies are warranted.

Currently, checkpoint inhibitors have been widely rationalized in several cancers for their great efficacy and accepted adverse effects compared with conventional therapies (36). If PD-L1 levels in tumor cells were linked to clinical and pathological features, the PD-L1 inhibitors would inhibit the tumor biology, such as invasion, recurrence, and metastasis, etc. In the study, PD-L1 was related to penile cancer in T stage, tumor grade, and LN status, similar findings have been indicated in both penile cancer and other types of tumors (37). Results indicated that PD-L1 could not promote the lymphovascular invasion (LVI) of penile cancer, while only three studies mentioned related data and some heterogeneity existed in these studies (17, 24, 26). The results may provide evidence for immunotherapy and rationalize it as a promising perioperative therapy for penile cancer.

Apart from PD-L1, some newly developed molecules related to genetic, epigenetic, and immune responses in patients with penile cancer should also be mentioned. Marchi et al. utilized data from a public database identifying *STAT1*, which is potentially associated with dysfunction of the immune system, as prognostic factor for penile cancer (38). Later, it was found that the knockdown of *CCL20*, *CXCL13*, or *CXCL5* gave the same results shown by significant suppression of *STAT1* level and inhibition of MMP2 and MMP9 expression, leading to the diminished proliferation, migration, and invasion of penile cancer cells (39, 40). *SHCBP1* is a gene that has physiological associations with T cell proliferation and signaling. Researchers have concluded that *SHCBP1* may be used as a prognostic biomarker. *In vitro* and *in vivo* experiments indicated that *SHCBP1* knockdown results in the decrease of the proliferation, migration, and invasion of penile cancer cells, while forced activation of *STAT3* reverses this process (41). Together, *SHCBP1* has the potential to be used not only as a biomarker but also as a target for future treatment strategies. Furthermore, more and more promising molecules and pathways, including *Pten*, *Smad4/Apc*, *PPARG, JAK–STAT*, and *MMP1* were found, providing potential therapeutic targets for the uncommon cancer (42, 43).

Our findings have some research and clinical implications. Firstly, the expression of PD-L1 may be a meaningful marker for prognosis anticipation in penile cancer cases. Patients with positive PD-L1 may tend to show more advanced tumor features and have a potentially worse cancer-specific prognosis. Secondly, PD-L1 expression maybe not the only biomarker associated with prognosis in the population, which should be identified in future laboratory studies. Thirdly, PD-1/PD-L1 blockades could be an effective treatment selection for penile cancer patients with positive PD-L1. We have also conducted a similar study in RCC (44). We found that the expression of PD-L1 in RCC is 27%, thus the expression of PD-L1 is more frequent in penile caner. Now that in the post-surgical treatment of advanced or metastatic renal cell carcinoma (RCC), immunotherapy has become the first-line drug (45). We believed PD-L1-related immunotherapy should have much more potential in the treatment of penile cancer. While the responsiveness of penile cancer to immunotherapy, just like other cancers, depends on several indicators, such as tumor mutation burden, immune infiltration patterns, macrophage infiltration patterns, and HPV status, etc. Future findings from clinical trials will hopefully elucidate the tangible conclusion regarding the role of immunotherapy in penile cancer. Given the rarity of the cancer, accruing patients to trials is difficult, suggesting the importance of multidisciplinary and global collaboration.

The study has some strengths. (A) The reliability of the results was repeatedly validated in the methodology. (B) IHC is an easy-to-use and widely-used method to evaluate the expression of protein. Therefore, the findings are clinically performable. The study also has some limitations. First and foremost, heterogeneity originating from the different cut-off values and sometimes the differences in primary antibody species, makes it difficult to reach a solid conclusion. Secondly, PD-L1 or PD-1 expressed by other cells or tissues were not evaluated and reviewed. Thirdly, four studies did not mention the staging standard (15, 22, 25, 26). Among other studies, those published after 2018 used different staging systems from those published before 2018. For studies after 2018, they even use different staging standards. This makes subgroup analysis unavailable. Thus, the differences in staging may cause bias, making the pooled results on stage unconvincing. Fourthly, we only roughly compared the usual SCC with all other unusual SCC. This was conducted and displayed because four of the included studies made this comparison and the pooled result was statistically calculatable. However, the clinical significance of the result is limited because unusual penile SCC consists of various subtypes and each subtype has its own pathological and prognostic features.

In conclusion, the positive rate of PD-L1 was 51.4% (95%CI: 42.1% to 60.8%) in penile cancer. Higher PD-L1 levels in tumor cells were related to shorter CSS in penile cancer patients and higher T stage, higher tumor grade, positive LN status, HPV negativity, and usual SqCC of penile cancer. Incorporating PD-L1 into prognostic tools for adjuvant treatment selection might help improve the survival of penile cancer. More studies comparing the efficacy difference and prognosis between patients with high PD-L1 and those with low PD-L1 are expected in the promising field.

## Data availability statement

The original contributions presented in the study are included in the article/**Supplementary Material**. Further inquiries can be directed to the corresponding author.

## Author contributions

(I) Conception: YL and HJL. (II) Administrative support: HJL. (III) Collection: YL, HS, and YTW. (IV) Data analysis and interpretation: HS and YTW. (V) Manuscript writing, revision and approval: All authors. All authors contributed to the article and approved the submitted version.

## Funding

This work is supported by the grant from National Population Health Science Data Sharing Service Platform Clinical Medical Science Data Center (NCMI-ABD02-201906) to HJL.

## Conflict of interest

The authors declare that the research was conducted in the absence of any commercial or financial relationships that could be construed as a potential conflict of interest.

## Publisher’s note

All claims expressed in this article are solely those of the authors and do not necessarily represent those of their affiliated organizations, or those of the publisher, the editors and the reviewers. Any product that may be evaluated in this article, or claim that may be made by its manufacturer, is not guaranteed or endorsed by the publisher.
