# Supplementary material for: PD-L1 is associated with the prognosis of penile cancer: A systematic review and meta-analysis
Source: Front Oncol. 2022 Nov 30;12:1013806. doi: 10.3389/fonc.2022.1013806 (PMC9748474; doi:10.3389/fonc.2022.1013806)
Supplement: Supplementary file 1 [file DataSheet_1.pdf]

Supplementary Material

1 Supplementary Table 1. Systematic search strategy (PICOS strategy).

Search strategy	
Population	#1 ((((((((((((((((((Penile Neoplasms[MeSH Terms]) OR (Penile Neoplasms)) OR (Penis Neoplasms)) OR (Neoplasm, Penis)) OR (Penis Neoplasm)) OR (Neoplasms, Penis)) OR (Neoplasms, Penile)) OR (Neoplasm, Penile)) OR (Penile Neoplasm)) OR (Cancer of Penis)) OR (Penis Cancers)) OR (Cancer of the Penis)) OR (Penis Cancer)) OR (Cancer, Penis)) OR (Cancers, Penis)) OR (Penile Cancer)) OR (Cancer, Penile)) OR (Cancers, Penile)) OR (Penile Cancers)
Intervention	#2 CD274 OR programmed cell death ligand 1 OR B7-H1 OR PD-L1 OR B7 homolog 1
Comparison	High vs. low expression of PD-L1 in tumor tissue
Outcomes	#3 Survival OR prognostic OR prognosis OR outcome
Study design	Randomized controlled trials, controlled clinical trials, prospective and retrospective cohort studies
Search combination	#1 AND #2 AND #3

Supplementary Table 2 The Newcastle-Ottawa scale (NOS) quality assessment of the included studies.

Study (first autor,year)	Study design	Selection	Comparability	Outcomes	Total
Udager 2016	RC	3	2	2	7
Cocks 2016	RC	3	2	3	7
Ottenhof 2016	RC	4	2	2	8
Deng 2017	RC	4	2	2	8
Davidsson 2018	RC	3	2	3	8
De Bacco 2019	RC	3	2	3	8
Chu 2020	RC	3	2	3	8
Hu 2020	RC	4	2	2	8
Müller 2022	RC	3	2	2	7

RC: retrospective cohort.
